# Photobiomodulation: shining a light on depression

**DOI:** 10.7150/thno.104502

**Published:** 2025-01-01

**Authors:** Lian Wang, Liwei Mao, Zhihai Huang, Jeffrey A. Switzer, David C. Hess, Quanguang Zhang

**Affiliations:** 1 Department of Neurology, Medical College of Georgia, Augusta University, 1120 15th Street, Augusta, GA, 30912, USA.; 2 Department of Neurology, Louisiana State University Health Sciences Center, 1501 Kings Highway, Shreveport, LA, 71103, USA.

**Keywords:** Photobiomodulation, Depression, Brain, Light, Mechanism

## Abstract

Depression is a prevalent public health issue, characterized by persistent low mood, impaired concentration, and diminished motivation. Photobiomodulation (PBM), which involves the application of red or near-infrared light, modulates physiological processes by enhancing cerebral blood flow, reducing inflammation, inhibiting apoptosis, and promoting neurogenesis. PBM can be administered transcranially or through systemic approaches, offering a potentially effective intervention for depression. This review discusses the characteristics of PBM, its underlying neurobiological mechanisms, and relevant physical parameters. Recent progress in both animal and clinical research underscores PBM's therapeutic potential for depression and emphasizes the need for further studies to establish a robust theoretical basis for standardized treatment protocols.

## 1. Introduction

### 1.1 Overview of depression

Depression is a widespread and debilitating mental health disorder that threatens physical and mental health globally and is a significant contributor to the rise of suicide in the 21st century. The COVID-19 pandemic has notably intensified the global prevalence of depression 1. A systematic review has revealed that the frequency of depressive symptoms persisting more than 12 weeks after COVID-19 infection ranged from 11% to 28% 2, with a marked increase also observed among healthcare professionals 3. Furthermore, post-COVID-19 syndrome, or long COVID, imposes considerable mental health burdens on individuals in recovery 4. In 2020, an estimated 221 million people globally were affected by depression 5, with around 17% of individuals projected to experience this condition at some point in their lives 6. Depression encompasses a spectrum of symptoms, such as social withdrawal, sadness, anxiety, apathy, sleep disturbances, changes in appetite, psychomotor retardation, and memory deficits 7. Among its forms, major depressive disorder (MDD) is distinguished by its persistence and intensity, often involving overwhelming hopelessness and a notable risk of suicidal ideation, making it one of the most severe depressive subtypes 8.

**Figure 1:**
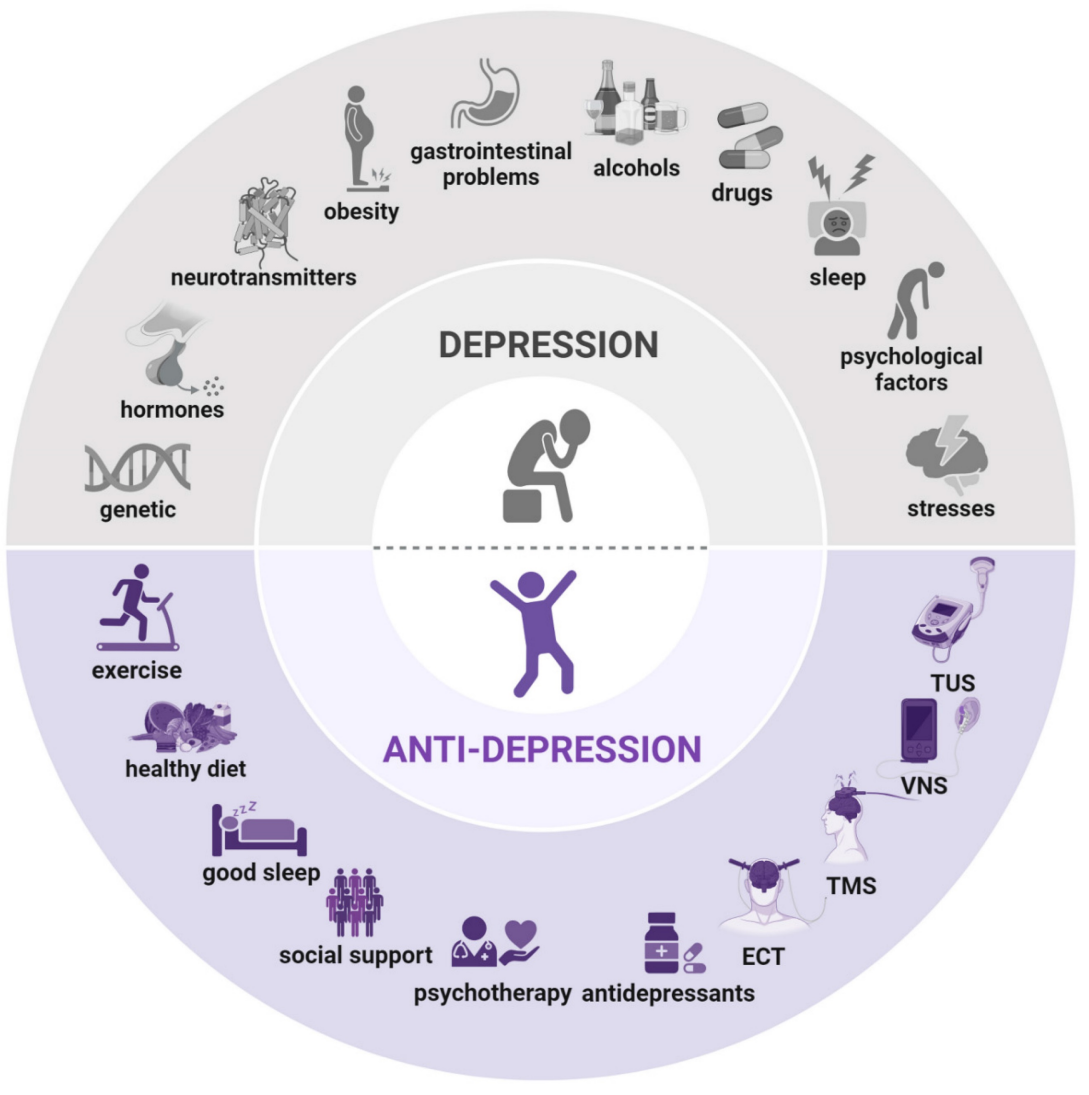
** Causes and treatment of depression. *Causes of depression (above)*:** genetic predisposition, hormonal imbalances, neurotransmitter dysregulation, obesity, gastrointestinal issues, alcohol consumption, drug use, sleep disturbances, psychological factors, and stresses. ***Treatment of depression (below)*:** exercise, balanced diet, adequate sleep, social support, psychotherapy, antidepressants, electroconvulsive therapy (ECT), transcranial magnetic stimulation (TMS), vagus nerve stimulation (VNS), and transcranial ultrasound stimulation (TUS). Created using BioRender.com.

The causes of depression involve a complex interplay of psychological, social, and epigenetic factors **(Figure 1)**. Physical pain and prolonged stress can trigger the onset of depression and influence its severity and progression 9. Extensive research indicates that sustained exposure to stress increases the risk of anxiety, depression, and other mood disorders, as well as metabolic syndrome, cardiovascular and cerebrovascular diseases, memory loss, cognitive impairment, learning difficulties, and structural changes in specific brain regions 10. Depression also exhibits variability in genetic influences, clinical course, neurobiological alterations, and treatment responses, often categorized into melancholic and atypical subtypes. Early detection of depression's underlying pathophysiology, potentially months or years before it reaches diagnostic thresholds, could mitigate patient suffering and reduce healthcare expenses 11.

Research into depression's pathogenesis suggests that both genetic predisposition and environmental stressors increase the risk of depressive disorders 12. A comprehensive understanding of depression's pathogenesis is therefore essential. To explore the molecular mechanisms underlying depression-like phenotypes, various animal models are employed in laboratory studies 13. While uniquely human experiences such as guilt, suicidal thoughts, and feelings of worthlessness cannot be replicated in animals, symptoms like anhedonia, social withdrawal, psychomotor slowing, neurotransmitter imbalances, and altered gene expression have been successfully modeled in rodents, often mirroring clinical observations in patients with depression **(Table 1)**.

### 1.2 Current treatments for depression

Conventional treatments for MDD primarily consist of pharmacological interventions and psychological therapy, achieving remission in only about 50% of cases 14. Although the development of various antidepressants has advanced mood disorder treatment, a significant proportion of patients remain unresponsive to current medications. Additional challenges include the delayed therapeutic onset of antidepressants and side effects that can impact adherence 15. Furthermore, relapse is common following initial remission. Evidence-based psychotherapy requires frequent sessions with trained mental health professionals, limiting accessibility 16.

For patients who cannot tolerate, are unresponsive to, or decline medication and psychotherapy, device-based treatments offer an alternative. Food and Drug Administration-approved options include electroconvulsive therapy, vagus nerve stimulation, and repetitive transcranial magnetic stimulation 17. Emerging experimental therapies, such as transcranial direct current stimulation, deep brain stimulation, magnetic seizure therapy, and transcranial ultrasound stimulation, are also under investigation** (Figure 1)**. Despite their efficacy, many patients with depression remain resistant to these neuromodulation therapies, which often involve complex procedures, including anesthesia, repeated clinical visits, and, in some cases, surgical implantation. Consequently, a safe, well-tolerated, effective, and convenient neuromodulation approach that patients can use independently at home would be highly beneficial.

### 1.3 Light therapy: a novel approach to treating depression

Light has been used therapeutically since ancient times, with early medical applications found in civilizations such as China, Egypt, and Greece 18. Approximately 3,500 years ago, Egyptians treated vitiligo by combining sunlight with extracts from the plant *Ammi majus*, native to the Nile River region, in a method that resembles modern photochemotherapy, formally introduced in 1974 and now applied to numerous diseases 19. Florence Nightingale later emphasized the importance of light in patient care, asserting that light, second only to fresh air, was essential for recovery, while darkness hindered healing. Her advocacy marked an early recognition of light's therapeutic role in health, supporting the use of both natural sunlight (heliotherapy) and artificial light (phototherapy) 20. In the 1850s, Arnold Rikli further promoted the healing benefits of light, establishing helio-hydrotherapy centers in Bled, Slovenia. A significant milestone followed when Niels Finsen demonstrated that ultraviolet light could effectively treat lupus vulgaris, a common skin disease of the era. His groundbreaking work earned him the Nobel Prize in Medicine in 1903, establishing a foundational achievement in phototherapy 21.

In 1960, Dr. Theodore Maiman's invention of the first practical laser advanced the field further 22. In 1967, Dr. Endre Mester discovered the laser's therapeutic potential, pioneering the use of low-level lasers to stimulate wound healing and hair growth in both animals and humans 23. Mester also observed systemic effects, noting therapeutic benefits even in areas distant from the treatment site. The introduction of affordable light-emitting diodes (LEDs) following early laser development further accelerated phototherapy's evolution. In the 1980s, Australian physiotherapist Louis Gifford advanced circadian rhythm research, establishing a basis for the role of photobiological regulation in health 24. Progress in circadian biology has since led to the application of bright light for conditions such as seasonal affective disorder 25. Sunlight exposure has additionally been shown to enhance cognitive function in patients with depression, with a notable dose-response effect 26. Overall, light, including natural sunlight, has been demonstrated to play a critical role in health and disease management, with evidence supporting its regulatory effects on numerous health conditions 27.

### 1.4 Photobiomodulation is a form of light therapy

Recent evidence suggests that photobiomodulation (PBM) may have significant therapeutic effects on health. PBM typically employs light sources within the visible (400-700 nm) and near-infrared (NIR) (700-1100 nm) ranges 28. Blue light (400-500 nm) penetrates the dermal layer, while green light (500-600 nm) can reach both the dermal and basal cell layers, primarily targeting superficial tissues. These wavelengths are commonly used for applications such as wound healing and anti-inflammatory treatments 29. Additionally, studies have begun to examine the effects of blue and green light on the osteogenic differentiation of stem cells *in vitro* 30. However, research in these areas remains in the preliminary stages, and some treatment parameters have reported adverse effects 31.

In contrast, red light (600-750 nm) and NIR light (750-1100 nm) have longer wavelengths that penetrate deeply, reaching several centimeters into muscle and other tissues, making them highly suitable for clinical applications targeting deep tissue structures. This review therefore focuses on red and NIR light in PBM, given their established efficacy in accessing deeper tissue layers and broad application in therapies for neurodegenerative diseases and trauma. Red and NIR spectra comprise a significant portion of Earth's solar energy and can also be produced through artificial light systems. PBM, also referred to as “low-level light therapy” or “low-level laser therapy,” employs red or NIR light from lasers or LEDs at low power densities to influence biological processes 32. Michael Hamblin demonstrated that PBM can stimulate, protect, repair, and regenerate damaged or degenerative tissue 33. Furthermore, PBM enhances stem cell activation, supporting cell migration, differentiation, proliferation, and neuronal survival, and modulates the activity of multiple protective genes 34. This mechanism makes PBM especially suitable for systems sensitive to energy demands, such as the nervous system.

**Figure 2:**
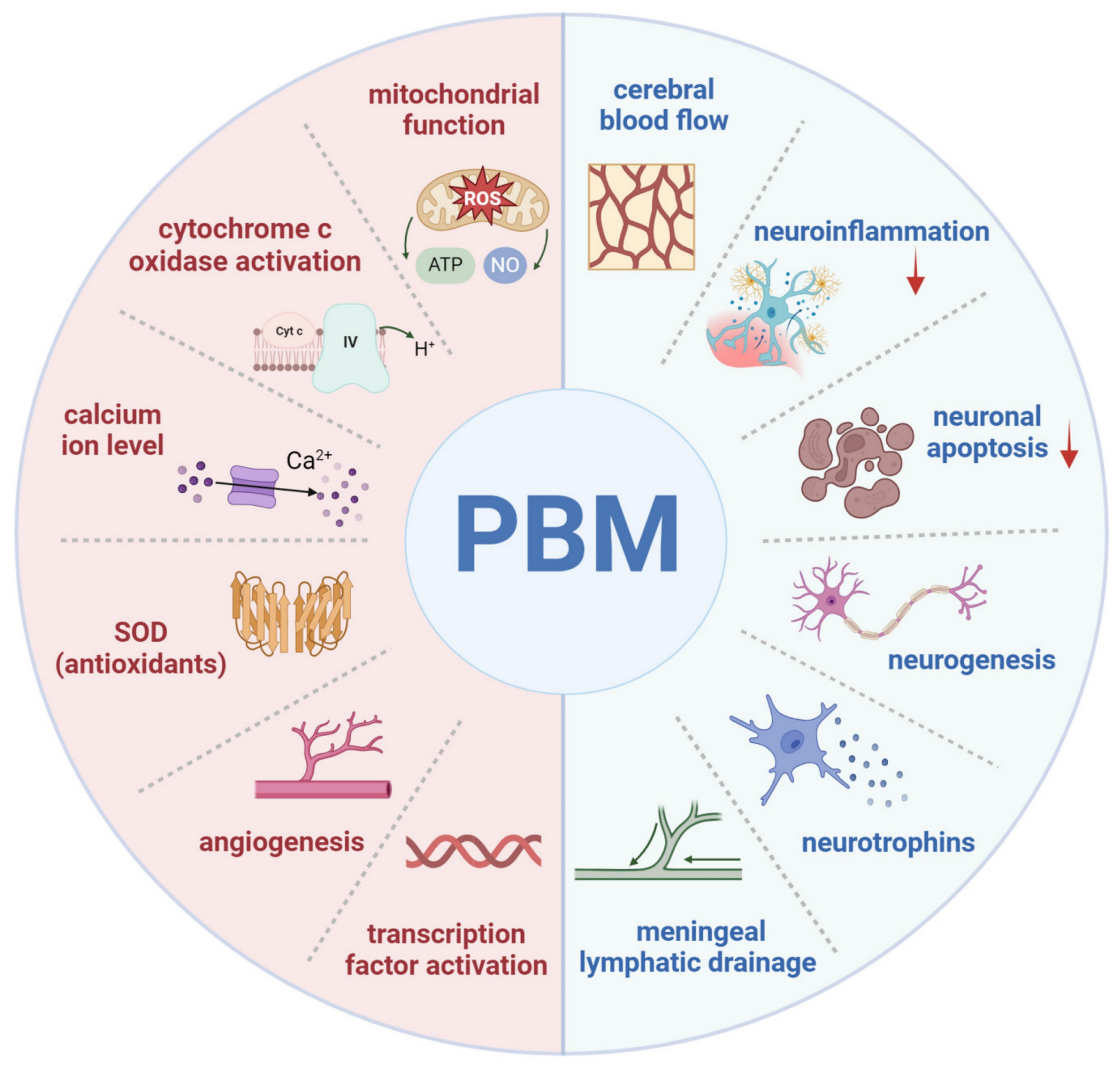
** Mechanisms and effects of photobiomodulation (PBM). *Cellular and molecular mechanisms of PBM (left)*:** enhancement of mitochondrial function, activation of cytochrome c oxidase, elevation of calcium ion levels, increased superoxide dismutase (SOD) (antioxidant) activity, promotion of angiogenesis, and activation of transcription factors. ***Neurophysiological effects of PBM (right)*:** enhancement of cerebral blood flow, modulation of neuroinflammation, reduction of neuronal apoptosis, promotion of neurogenesis, increased production of neurotrophins, and facilitation of meningeal lymphatic drainage. Created using BioRender.com.

PBM typically uses reflective devices to deliver controlled low-energy light, with a safe dose range of 4-30 J/cm² and 5-50 mW/cm², applying red to NIR wavelengths to brain tissue 35. Following confirmed efficacy in treating acute stroke, PBM has expanded to broader applications in brain therapies 36. Subsequent research has demonstrated numerous benefits, including the repair of central nervous system damage, improvement of cerebral blood flow (CBF), stimulation of neurogenesis in neurons and glial cells, promotion of synaptogenesis and cell migration, and elevation of brain-derived neurotrophic factor (BDNF) levels **(Figure 2)** 37. PBM has also shown effectiveness in treating traumatic brain injury (TBI) and neurodegenerative disorders 38, 39. As a result, PBM has emerged as an innovative and promising approach in neurological therapies.

Multiple studies have investigated the use of PBM as a treatment for neuropsychiatric disorders 40. In the field of psychology, PBM has demonstrated benefits for cognitive functions, including learning, memory, executive function, attentional control, and mental health disorders 41. Depression is associated with various structural and molecular alterations in the brain, such as hippocampal atrophy, cell death in the dentate gyrus, reduced connectivity between the hippocampus and prefrontal cortex, disruption of brain circuits, weakened neuronal networks, and mitochondrial dysfunction 42. Furthermore, reduced prefrontal CBF is frequently observed in depression and other mental disorders 43. Additional findings indicate increased oxidative stress, neuroinflammation, apoptosis, hypometabolism, and decreased levels of BDNF 44. A potential link has also been suggested between biophotons and depression, as biophoton emission intensity may reflect oxidative stress and reactive oxygen species (ROS) production. Consequently, PBM is considered a promising alternative for targeting the dysfunctional brain mechanisms underlying depression. PBM, therefore, holds promise as an innovative, non-invasive, cost-effective therapeutic approach for neurological, psychological, and psychiatric conditions.

Preclinical studies have examined various aspects of PBM brain therapy, including safety and optimal treatment parameters, such as wavelength, fluence, irradiance, number of sessions, treatment duration, and light delivery mode (continuous or pulsed) 45. Current research on PBM remains in its early stages; although beneficial effects have been consistently observed in preclinical studies, substantial clinical research is needed to validate these findings in humans. Preliminary results suggest PBM's potential as an effective therapy for numerous neurological disorders, including neurotrauma (stroke, TBI), neurodegeneration (Alzheimer's disease (AD), Parkinson's disease (PD)), and neuropsychiatric conditions (depression, anxiety). Additionally, PBM shows promise as a method for cognitive enhancement in healthy individuals 46. With its affordability, safety profile, and ease of use, PBM is well-suited for at-home applications and could become widely accessible. As a novel device-based neuromodulation technique, PBM has demonstrated antidepressant effects in both animal and human studies 47. Nevertheless, its clinical application remains contentious, as study outcomes vary and treatment parameters are still being optimized. This review aims to summarize current research on PBM's neuroprotective effects in depression, with a focus on evaluating its biological mechanisms, neurobiological impact, physical parameters, and therapeutic potential.

## 2. Methods

A systematic search was conducted across PubMed, Web of Science, and Embase databases, focusing on English-language publications from January 2014 to September 2024. The search targeted studies investigating the mechanisms of PBM therapy for depression, including both preclinical and clinical research. Key search terms included "photobiomodulation," "red light," "near-infrared light," "LED," "laser," "low-level light therapy," "depression," "mental health," "mood disorders," "brain," "transcranial," "wavelength," "fluence," "irradiance" "cytochrome c oxidase," and "mechanism." Additionally, ClinicalTrials.gov was reviewed to identify ongoing and completed clinical trials relevant to recent advancements in PBM applications for depressive disorders. Reviews lacking original data were excluded, as were studies that solely reported on original animal research, clinical studies, or ongoing clinical trials not yet published in peer-reviewed journals.

## 3. Cellular and molecular mechanisms of PBM

### 3.1 Mitochondrial function and cytochrome c oxidase

Light can activate chromophores within biological tissues, enhancing enzyme activities in the mitochondrial electron transport chain, including complexes I-IV and succinate dehydrogenase. This chain facilitates electron transfer from cytochrome c to molecular oxygen across the inner mitochondrial membrane, accompanied by multiple redox reactions. As the terminal enzyme in this chain, cytochrome c oxidase (CCO), or “complex IV,” is considered a primary chromophore that absorbs light within the red to NIR spectrum **(Figure 2)**. Exposure to red and NIR light through PBM increases electron availability at the CCO catalytic site, enhancing molecular oxygen reduction. This process leads to an increase in mitochondrial membrane potential (MMP), alongside elevated levels of ROS, adenosine triphosphate (ATP), nitric oxide (NO), calcium ions (Ca²⁺), and cyclic adenosine monophosphate (cAMP). Collectively, these changes reflect enhanced mitochondrial function and an elevated cellular metabolic rate 34.

**Figure 3:**
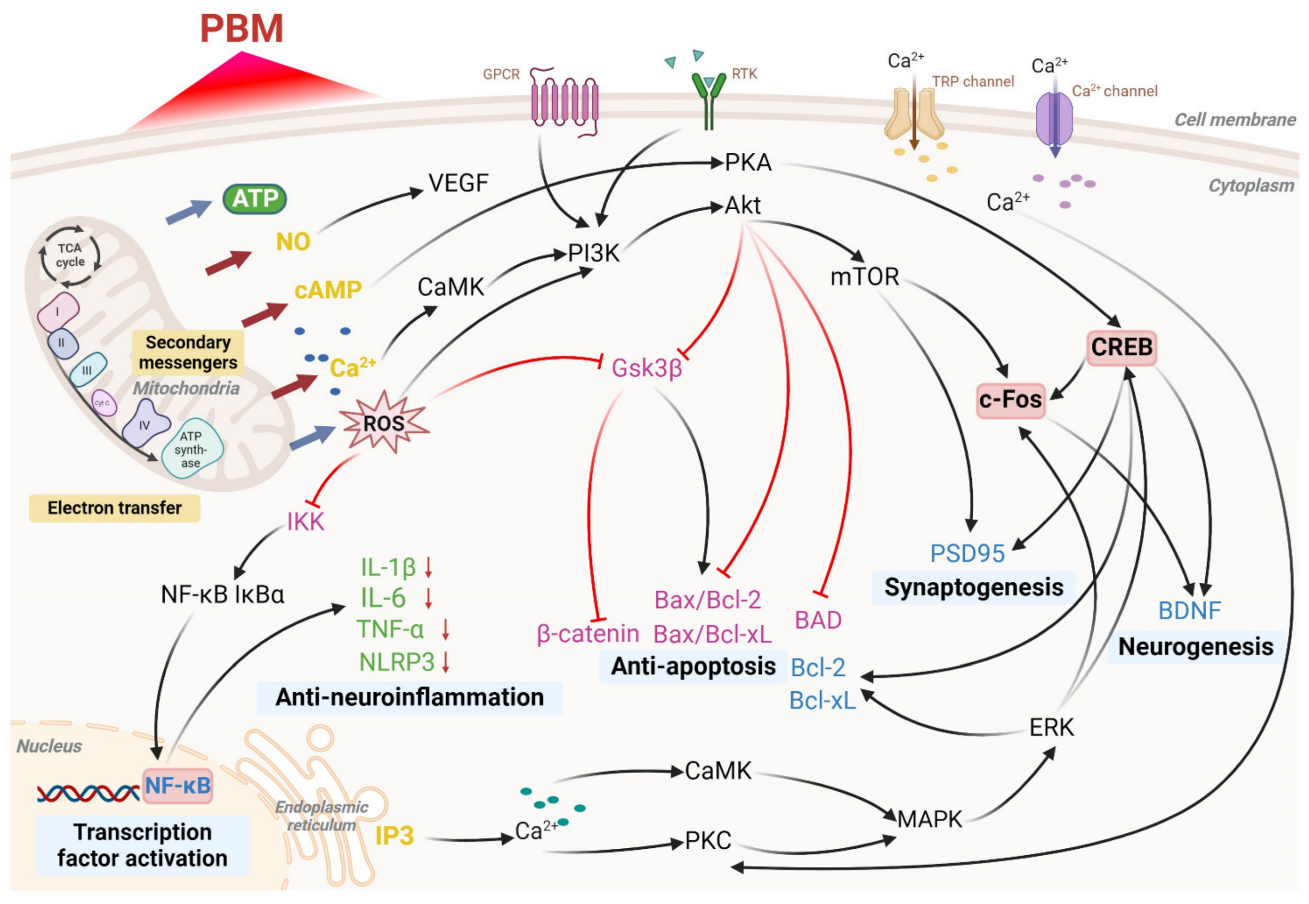
** Molecular pathways activated by photobiomodulation (PBM).** PBM influences mitochondria, cell membrane receptors, calcium ion channels, and stimulates intracellular calcium release from the endoplasmic reticulum, resulting in increased production of adenosine triphosphate (ATP) and reactive oxygen species (ROS), nitric oxide (NO) release, elevated levels of cyclic adenosine monophosphate (cAMP), and enhanced calcium ion (Ca²⁺) signaling. These processes activate a series of cellular pathways that ultimately regulate transcription factor activation, neuroinflammation reduction, apoptosis inhibition, synaptogenesis, and neurogenesis. Abbreviations: Protein Kinase B, Akt; Bcl-2-associated death promoter, BAD; brain-derived neurotrophic factor, BDNF; B-cell lymphoma 2, Bcl-2; B-cell lymphoma-extra large, Bcl-xL; calcium/calmodulin-dependent protein kinase, CaMK; cAMP response element-binding protein, CREB; extracellular signal-regulated kinase, ERK; G-protein-coupled receptor, GPCR; glycogen synthase kinase 3 beta, Gsk3β; IκB kinase, IKK; interleukin 1 beta, IL-1β; interleukin 6, IL-6; inhibitor of kappa B alpha, IκBα; inositol 1,4,5-trisphosphate, IP3; cytochrome c oxidase (complex IV), IV; mitogen-activated protein kinase, MAPK; mechanistic target of rapamycin, mTOR; nuclear factor-κB, NF-κB; NOD-like receptor family pyrin domain containing 3, NLRP3; phosphoinositide 3-kinase, PI3K; protein kinase A, PKA; protein kinase C, PKC; postsynaptic density protein 95, PSD95; receptor tyrosine kinase, RTK; tricarboxylic acid cycle, TCA cycle; tumor necrosis factor alpha, TNF-α; transient receptor potential, TRP; vascular endothelial growth factor, VEGF. Created using BioRender.com.

Following these initial mitochondrial responses, various signaling cascades are activated, ultimately inducing transcription factors **(Figure 3)**. *In vitro* studies have demonstrated that red and NIR light in the 600-860 nm range with an influence of 52 J/m^2^ enhances HeLa cell adhesion. This effect is thought to involve interactions between the mitochondria, plasma membrane, and nucleus, as inhibition of CCO, Na⁺/H⁺ exchangers, or the Na⁺/K⁺-ATPase pump prior to light exposure eliminates the enhancement of cell attachment 48. Studies in animal models suggest that PBM can achieve antidepressant effects comparable to pharmacological interventions, primarily by stimulating mitochondrial metabolism through CCO activation upon red and NIR light absorption 49. In studies involving elderly participants, 670 nm light treatment has been shown to significantly increase ATP synthase activity in the brain, suggesting that PBM may enhance mitochondrial function and brain energy metabolism, potentially supporting improved health outcomes 50.

### 3.2 Nitric oxide

As an endogenous vasodilator, NO plays a vital role in maintaining normal circulation. NO binds to CCO, displacing oxygen; in excessive amounts, however, NO can inhibit mitochondrial respiration 51. PBM may counteract this inhibitory effect by enhancing CCO activity through the photodissociation of NO from CCO **(Figure 2)**. During this process, NO is photolyzed from the copper and heme iron centers where it is bound, allowing oxygen to rebind and restoring CCO activity, thus facilitating oxygen influx and the resumption of normal cellular respiration 52. Light exposure can also prompt the release of NO from various intracellular sites, such as nitrosylated proteins like myoglobin and hemoglobin, resulting in similar beneficial effects. The NO released by photodissociation exerts vasodilatory effects and enhances lymphatic circulation. In addition, PBM promotes the activity of endothelial nitric oxide synthase (eNOS), increasing NO production, which improves endothelial function and supports vascular homeostasis, particularly in conditions of eNOS dysfunction 53. NO also functions as a potent signaling molecule, activating various protective cellular pathways **(Figure 3)**. Relevant studies have shown that transcranial PBM (tPBM) using an 810 nm laser can prevent anxiety and depression in mouse models by modulating serotonin and NO levels in the brain, with an optimal dose of 8 J/cm² 54.

### 3.3 Oxidative stress

ROS are primarily generated within mitochondria and are closely linked to oxidative stress. Oxidative stress occurs when the body's antioxidant defenses are insufficient to counteract ROS production, allowing ROS to exert damaging effects when present in excess over prolonged periods 55. The therapeutic or detrimental outcomes of PBM are partially influenced by the quantity of ROS generated by mitochondria **(Figure 2)**. Research indicates that photon absorption by mitochondria during PBM induces a transient increase in ROS 56. This ROS surge is thought to activate mitochondrial signaling pathways that confer antioxidant, anti-apoptotic, and cytoprotective effects within cells. As noted, low doses of PBM stimulate minimal mitochondrial ROS production, which contributes to the modulation of cellular signaling pathways **(Figure 3)**. Conversely, higher PBM doses can lead to excessive ROS generation, initiating apoptotic pathways 57. Given that mitochondria serve as primary interaction sites for red and NIR light in cellular environments, cerebral PBM is considered an initial intervention for reversing mitochondrial dysfunction induced by oxidative stress. Several studies have explored PBM's role in regulating ROS levels and its potential impact on the pathophysiology of depression, suggesting that PBM may alleviate depressive symptoms by modulating oxidative stress and cellular signaling pathways 58.

### 3.4 Intracellular Calcium homeostasis and signaling

PBM regulates intracellular calcium levels through various mechanisms, resulting in a wide range of biological effects** (Figure 2)**. PBM enhances mitochondrial function by increasing CCO activity, which subsequently alters the MMP. This change affects Ca²⁺ flux across the mitochondrial membrane, leading to elevated intracellular Ca²⁺ levels 59. Activation of CCO by PBM boosts ATP production and its release, which can subsequently stimulate purinergic P2X receptors, facilitating Ca²⁺ influx through calcium channels 60. Additionally, PBM may further influence Ca²⁺ levels by activating transient receptor potential channels, initially identified in *Drosophila* and known to function as Ca²⁺ channels in response to light, heat, and other stimuli 61. PBM can also promote Ca²⁺ release from intracellular stores, such as the endoplasmic reticulum, by regulating secondary messengers like inositol 1,4,5-trisphosphate (IP3), further increasing intracellular Ca²⁺ levels 62. This elevation in intracellular Ca²⁺ is thought to contribute to several beneficial effects of PBM, including enhanced cellular repair, reduced inflammation, and improved signal transduction via pathways such as calmodulin kinase, cAMP/PKA, PKC, and Ras/ERK **(Figure 3)** 63.

### 3.5 Other signaling pathways

Research suggests that the beneficial effects of transcranial tPBM on brain function result from enhanced oxygen supply and utilization, improved mitochondrial function, and increased ATP synthesis **(Figure 2)** 64. Numerous studies have documented that even brief light exposure, particularly in experimental models of acute injury or trauma, can produce effects that persist for extended durations 65. Our previous study investigated the role of PBM in a hypoxia-ischemia rat model, demonstrating that PBM effectively reduces neuroinflammation, protects neurons, and facilitates recovery following ischemic injury 66. Moreover, it has been shown that PBM application in rats with repeated closed-head injuries significantly improved motor performance, alleviated anxiety, and reduced cognitive deficits, while also promoting neuronal survival and minimizing nerve damage 67. Subsequent *in vitro* and *in vivo* studies revealed that PBM supports neuronal protection in stroke models by promoting synaptic integrity and reducing stroke-induced damage through inhibition of neurotoxic astrocyte polarization 68. An additional *in vitro* study showed that PBM at a 660 nm wavelength (2.4, 3.21, and 5.35 J/cm²) promotes cell proliferation in CCO-deficient cell lines, suggesting that PBM's biological effects may not be entirely dependent on CCO and indicating the potential for other targets of laser photons 69.

These sustained effects of PBM may not be entirely explained by mitochondrial activity alone but rather by its activation of signaling pathways and transcription factors, resulting in prolonged alterations in protein expression **(Figure 3)**. A recent study identified at least fourteen distinct transcription regulators and signaling molecules activated by light exposure 34. Numerous studies have also examined PBM's role in regulating key signaling pathways associated with depression, highlighting its potential to alleviate depressive symptoms by modulating neuroinflammation, oxidative stress, and related cellular signaling mechanisms 49.

## 4. Neurophysiological effects of PBM

### 4.1 Cerebral blood flow

Reduced CBF is often one of the earliest indicators of various neurological conditions 70. Through photodissociation, PBM can release NO, a potent vasodilator, from its binding sites within the mitochondrial electron transport chain. Animal studies have demonstrated that PBM can elevate neuronal NO levels, expand blood vessel diameter, as well as enhance CBF **(Figure 2)** 71. Several investigations have examined the ability of PBM to modulate regional CBF. For instance, an animal study reported a 30% enhancement in CBF following NIR laser exposure 72. Research by Dias *et al.* revealed dose-dependent modulation of vascular endothelial growth factor (VEGF) and its corresponding receptor VEGFR-2 in masseter muscles of rats after ten sessions of 780 nm (2.5, 5.0, and 20 J/cm²) laser exposure **(Figure 3)** 73. Another study found that 1064 nm PBM (15 J/cm²) preconditioning enhanced CBF and improved ischemic stroke prognosis in mice by promoting eNOS phosphorylation, highlighting PBM's potential as a non-invasive adjuvant therapy for stroke 74. In addition, repeated exposure to NIR LEDs in an individual with a prolonged vegetative state increased local blood perfusion in the forehead by 20% 75. A larger clinical trial involving 25 healthy elderly females found that local CBF was elevated through transcranial LED treatment with a red-wavelength spectrum 76. Therefore, these findings suggest that targeting specific brain regions with PBM may modulate regional CBF. Research indicates that PBM can alleviate depressive symptoms by enhancing CBF, thereby improving oxygenation and nutrient delivery to brain regions associated with emotional regulation 77.

### 4.2 Neuroinflammation

Inflammation serves as a primary defense mechanism of the innate immune system in response to external agents such as viruses and bacteria. Neuroinflammation, a critical pathophysiological feature of various brain disorders, is predominantly mediated by activated microglial cells 78. Microglia release inflammatory mediators, such as cytokines and chemokines, and undergo morphological and functional changes that can lead to different forms of neuronal injury 79. Excessive ROS production promotes the translocation of the transcription factor nuclear factor-κB (NF-κB) into the cell nucleus, subsequently triggering the production of inflammatory signaling molecules 80. However, PBM has been shown to modulate cytokine levels, which play a central role in immune response signaling. PBM has been reported to regulate both pro-inflammatory and anti-inflammatory cytokines **(Figure 2)** 81. Low-dose PBM generates a controlled amount of ROS, inhibits NF-κB and other signaling pathways, and ultimately alleviates inflammatory responses 82.

In tPBM research, tumor necrosis factor-alpha and specific interleukins (IL), particularly IL-1β and IL-6, are the most extensively studied cytokines **(Figure 3)** 83. Research has demonstrated that 780 nm tPBM (20 J/cm²) reduces cerebral infarct size, modulates microglial activation, and decreases neuroinflammatory responses in stroke models, demonstrating neuroprotective effects 84. Moreover, 808 nm tPBM (133.3 J/cm²) significantly inhibited neuroinflammation, astrogliosis, and microgliosis in the hippocampus of adolescent rats, thereby reducing the severity of pentylenetetrazole-induced seizures 85. In a mouse model of autism spectrum disorder induced by prenatal valproic acid exposure, 830 nm PBM (42 J/cm^2^) reduced cognitive dysfunction and neuroinflammation 86. PBM has also been reported to relieve depression by specifically targeting and downregulating neuroinflammatory pathways, thereby lowering proinflammatory cytokine levels and mitigating the neurological damage associated with depressive symptoms 87.

### 4.3 Neuronal apoptosis

Apoptosis is a key pathophysiological process involved in both normal brain aging and neurodegenerative disorders such as AD and PD 88. The mitochondria-mediated pathway, also known as the intrinsic pathway, represents a crucial mechanism of programmed cell death. This pathway is initiated by a reduction in MMP and the release of cytochrome c from the mitochondria and into the cytoplasm 89. In the cytoplasm, cytochrome c interacts with Apaf-1 to form the apoptosome, a complex that subsequently activates caspase-9, leading to the activation of caspase-3 and ultimately resulting in apoptosis 90. The Bcl-2 family, comprising both pro-apoptotic and anti-apoptotic members, plays a critical role in regulating apoptosis. Apoptosis is often triggered by Bax overexpression or an elevated Bax/Bcl-2 ratio, leading to activation of the caspase cascade 91. Studies have shown that PBM reduces apoptosis induced by subchronic restraint stress, evidenced by decreased Bax/Bcl-2 and cytosolic/mitochondrial CCO ratios 92.

In addition to its effects on the Bcl-2 family, PBM has been shown to interact with other signaling pathways involved in apoptosis regulation. For instance, the PKC family of serine/threonine kinases influences apoptosis by modulating levels of Bax and Bcl-xl, with PKC activation ultimately inhibiting apoptosis 93. Low-dose laser treatment at 0.156, 0.312, and 0.624 J/cm² has been shown to reduce the Bax/Bcl-xl ratio via PKC pathway activation, effectively reversing apoptosis in PC12 cells 94. Further research has demonstrated that PBM attenuates Aβ-induced apoptosis by activating the Akt signaling pathway, inhibiting glycogen synthase kinase 3 beta, and stabilizing β-catenin, highlighting its potential as a therapeutic approach for neurodegenerative diseases 95. Additional studies have reported a reduction in the Bax/Bcl-2 ratio following tPBM intervention **(Figure 3)** 96. Moreover, PBM has been shown to alleviate depressive symptoms by reducing neuronal apoptosis, underscoring its role in modulating apoptotic pathways and preventing neuronal loss associated with depression **(Figure 2)** 92.

### 4.4 Neurogenesis

The promotion of synaptogenesis and neurogenesis by PBM is among its most notable and potentially impactful effects on the brain **(Figure 2)**. PBM has been reported to enhance the expression of BDNF, nerve growth factor, and glial cell line-derived neurotrophic factor—key members of the neurotrophin family that have garnered considerable interest 97. The triggering of internal IP3 receptors by PBM with 632.8 nm light (0.5, 1, 1.9, and 3.8 J/cm^2^) resulted in increased intracellular Ca^2+^ levels, which in turn activated the ERK/CREB pathway and ultimately led to improved BDNF expression 98. *In vivo* studies have demonstrated that coherent 670 nm laser light (4 J/cm^2^) significantly increased BDNF expression in the rat occipital cortex 99. PBM positively influences dendritic morphogenesis and neuronal connectivity by stabilizing BDNF levels through the ERK/CREB signaling pathway 100. BDNF enhances synaptogenesis by mediating synapsin 1, a downstream factor that promotes neuronal fiber development and preserves synaptic contact 101.

Beyond synaptogenesis, PBM contributes to neurogenesis by increasing levels of IFN-γ and IL-10 in CD4^+^ T cells and upregulating postsynaptic density protein 95 expression, which supports synaptogenesis and enhances cognitive function in an AD mouse model **(Figure 3)** 102. In another study, tPBM at 810 nm (8 and 16 J/cm^2^) inhibited inflammatory factor production, increased synaptophysin expression, upregulated synaptic markers, and improved cognitive function in an aged mouse model 103. In a recent primate PD model, intracranial PBM treatment with non-coherent 670 nm LED light increased glial cell line-derived neurotrophic factor levels in the striatum, leading to behavioral improvements 104. Infrared light exposure, whether acute or chronic, was also found to elevate the number of BrdU-labeled cells in the hippocampal CA1 region, indicating enhanced cell proliferation 105. In a stroke animal model, PBM reduced infarct size and increased biomarkers associated with cell proliferation 106. Deficits in neurogenesis are implicated in the pathogenesis of depression; mice with neurogenesis impairments exhibit elevated corticosterone levels and increased depressive-like behaviors following acute stress 107. Conversely, enhancing adult hippocampal neurogenesis has been shown to alleviate anxiety and depressive behaviors in chronically stressed mice 108. Therefore, it is plausible that enhanced neurogenesis is a key mechanism underlying PBM's antidepressant effects.

## 5. Physical parameters involved in PBM

### 5.1 Biphasic dose response

The biphasic dose-response is a fundamental biological principle, demonstrating that low doses of physical or chemical agents can activate or stimulate various biological systems, even if these agents may be toxic or harmful at higher doses 109. Numerous studies have shown that PBM follows the Arndt-Schulz curve, exhibiting a biphasic dose-response 110. Achieving therapeutic outcomes relies on delivering an appropriate light dose to the target tissue. While PBM provides benefits at lower doses, these effects diminish as the dose increases, ultimately resulting in harmful outcomes at very high doses 111. The biphasic response of PBM is supported by a range of *in vitro* and *in vivo* studies 112.

Research suggests that excessive doses of PBM may produce suppressive effects 113. For instance, studies on cultured cortical neurons demonstrated that the highest ATP production efficiency, along with increased MMP and Ca²⁺ levels, occurred at a steady intensity of 25 mW/cm² with a dose of 3 J/cm² 114. In contrast, lower doses of 0.03 and 0.3 J/cm², as well as a higher dose of 10 J/cm², produced only minor stimulatory effects, while a fluence of 30 J/cm² resulted in suppressive effects due to mitochondrial impairment. Optimal parameters for transcranial stimulation to treat MDD remain undetermined. The variability in parameters, targeted regions, and treatment protocols in clinical research on tPBM for MDD makes it challenging to reach definitive conclusions regarding dose-response relationships. These studies mainly focus on parameters such as wavelength, fluence, irradiance, and exposure duration.

### 5.2 Light penetration

Various optical parameters influence light penetration in tissues, including wavelength, fluence, irradiance, pulse structure, coherence, and polarization 115. In biological tissues, light energy is reduced through scattering and absorption by biomolecules, impacting the amount of energy reaching the cerebral cortex. The human cranial bone, consisting of water (12.2%), carbohydrates (5.2%), protein (24.6%), and minerals (58%), demonstrates high levels of optical absorption and scattering 116. Human skulls are notably thicker than those of species commonly used in preclinical studies, such as mice, rats, and rabbits. Comparative studies on 800 nm light transmission across cranial bones of various species found that the human skull transmitted only 4.18-4.24% of the light, markedly lower than transmission rates observed in mice (40.10%), rats (21.24%), and rabbits (11.26%) 117. Research by Jagdeo *et al.* using human cadaver heads demonstrated that 830 nm light transmission varied by skull region, with 0.9% transmission through the temporal area, 2.1% through the frontal area, and 11.7% through the occipital area 118.

#### 5.2.1 Wavelengths

Although PBM using red and NIR light spans wavelengths from 600 to 1100 nm, certain wavelengths are specifically associated with mitochondrial activity 119. Oxidized CCO absorbs light within the 600-680 nm and 800-870 nm spectral bands, whereas reduced CCO absorbs light within the 730-770 nm band 120. Notably, the absorption peaks of oxidized CCO correlate directly with action spectra observed in biological processes such as DNA synthesis 121. NIR light at 810 nm activates CCO, enhances mitochondrial oxygen consumption, and increases ATP production 122. Comparative studies on the 810 nm and 980 nm wavelengths suggest that 810 nm (3 J/cm^2^) affects CCO activity, while 980 nm (0.03, 0.3 J/cm^2^) influences temperature-sensitive Ca²⁺ channels 123. Additionally, low-level NIR-II (1064 and 1270 nm) has been shown to significantly enhance NO availability in endothelial cells 124. Studies demonstrate that 660 nm PBM (3 J/cm^2^) effectively reduces oxidative stress and enhances BDNF expression in the hippocampus of mice and hippocampal cell lines, suggesting neuroprotective potential 125. In an* in vitro* blood-brain barrier model, 808 nm NIR light (10 and 30 J/cm^2^) was found to increase blood-brain barrier permeability, indicating potential for improved drug delivery to the brain 126.

Evidence further indicates that 810 nm PBM (33.3 J/cm^2^) can reduce cognitive impairment and improve neurological outcomes in artificially aged mice with transient cerebral ischemia, as well as in mice with controlled cortical impact and closed-head TBI, underscoring its role in mitigating ischemic and traumatic brain damage 127. Notably, the use of NIR-II (1000-1700 nm) is emerging as a promising therapeutic tool, offering deep tissue penetration, reduced scattering, and minimal tissue absorption 128. Research highlights the differential effects of PBM wavelengths (800, 850, and 1064 nm) and light source types (laser and LED) on mitochondrial redox metabolism and hemoglobin oxygenation. The 810 nm LED shows a distinct and more rapid dynamic response, whereas the 1064 nm laser is associated with a prolonged effect 129. A recent study indicated that 1064 nm laser (15 J/cm^2^) pretreatment in stroke models elevates eNOS phosphorylation and increases CBF, thereby improving stroke prognosis 74. Human studies further indicate that tPBM with a 1064 nm wavelength laser enhances metabolic activity in the brain, suggesting potential benefits for cognitive function and overall brain health 130. Additionally, 1267 nm PBM (32 J/cm^2^) has shown promise in enhancing meningeal lymphatic drainage, clearing β-amyloid from the brain, and improving cerebral oxygen saturation in mice, representing a potential therapeutic strategy for AD by supporting lymphatic function 131.

#### 5.2.2 Fluence and irradiance

Light can penetrate approximately 1 to 3 cm through the skin, cranial bone, cerebrospinal fluid, and brain membranes to reach the human prefrontal cortex effectively 132. The actual dose reaching the cortex depends on both the total energy transmitted to the scalp and the fraction that penetrates into the brain. Therefore, it is essential to deliver sufficient energy to the skin surface to ensure that an adequate and safe fluence reaches target brain regions. Fluence, defined as the energy delivered per square centimeter (J/cm²), is calculated by multiplying irradiance (mW/cm²) by exposure duration (seconds). PBM on neural cells has been shown to be effective at fluence levels between 0.1 and 15 J/cm² 133. In human tPBM studies, fluence applied to the head has varied depending on the condition: ranges of 10 to 30 J/cm² for neurological disorders 134, 13 to 84 J/cm² for psychological conditions 135, and 15 to 60 J/cm² for studies involving healthy subjects 136. Additionally, the effectiveness of PBM therapy is closely linked to the dosage administered and the initial severity of the condition, with both factors contributing significantly to the overall therapeutic outcomes.

#### 5.2.3 Operation mode

PBM therapy has been administered using either continuous wave or pulsed wave at repetitive frequencies ranging from 1 to 3000 Hz 137. Several studies have supported the enhanced safety and biostimulatory benefits of pulsed wave over continuous wave 138. The pulsed wave mode limits light exposure by increasing peak irradiance and reducing skin heating, allowing for greater penetration than continuous wave. It has been reported that pulsed wave results in reduced heat production and no tissue injury, whereas continuous wave has been associated with neurological impairments and histopathological damage due to thermal effects 45. A human study observed that pulsed tPBM at 40 Hz and 100 Hz in the Gamma band led to significant cognitive improvements and more pronounced changes in brain electrical activity 139. In a recent study, Tsai *et al.* employed multisensory 40 Hz gamma stimulation, combining bright light and sound in a 5×FAD AD mouse model, to significantly enhance cerebrospinal fluid inflow and interstitial fluid outflow, thereby promoting amyloid clearance through the glymphatic system 140. Additionally, they demonstrated that this stimulation significantly reduced cuprizone-induced demyelination, increased oligodendrocyte production, and decreased neuroinflammation in male mice 141.

Beyond cognitive benefits, a 40 Hz light flash has demonstrated efficacy in treating insomnia 142. While these studies do not fall strictly under PBM, they underscore the importance of pulsed light in therapeutic stimulation. Moreover, 10 Hz pulsed laser therapy has demonstrated effectiveness across various models: promoting skin wound healing in immunosuppressed rats, reducing inflammation and brain damage in TBI mice, enhancing mitochondrial function and neurogenesis, and mitigating Aβ accumulation and cognitive impairment in AD mice through microglial modulation 143. Pulsed wave therapy has also shown promise in patients with neurodegenerative diseases 144. Notably, both animal and clinical studies have observed antidepressant effects with 10 Hz pulsed wave 145. Further research in the PBM field is warranted to explore the specific effects of the pulsed operation mode.

#### 5.2.4 Light sources

PBM therapy commonly employs both laser and LED devices, with LED therapy proving as effective as laser therapy for superficial tissues. Power densities vary significantly, with lasers typically requiring active cooling at approximately 700 mW/cm², while LEDs operate at lower power densities, typically between 10 and 30 mW/cm² 146. Lasers deliver higher energy to small treatment sites than LEDs, raising concerns regarding heat generation and potential tissue damage. LEDs, on the other hand, offer a broader range of total power levels (especially useful for treating frontal regions), determined by the size of the array and the number and power of individual diodes. Due to their spatial divergence, LEDs can cover a larger area simultaneously, are more cost-effective, and are convenient for home use 147.

In terms of light quality, coherent monochromatic lasers provide greater light coherence compared to non-coherent LEDs 148. LEDs generally produce unpolarized light, which is more susceptible to scattering in tissues, potentially reducing precision and depth of penetration. In contrast, lasers produce coherent, polarized light, enhancing targeting accuracy and enabling deeper tissue penetration, which may be more effective in certain PBM applications 149. Research on polarized PBM has shown enhanced cell viability and proliferation, improved MMP, and reduced apoptosis in human fibroblasts, suggesting polarized PBM as a promising approach for promoting wound healing 150. Although polarized light therapy has demonstrated promising biological effects, further research is required to fully understand and validate its potential applications in phototherapy.

## 6. Types of PBM therapy

**Figure 4:**
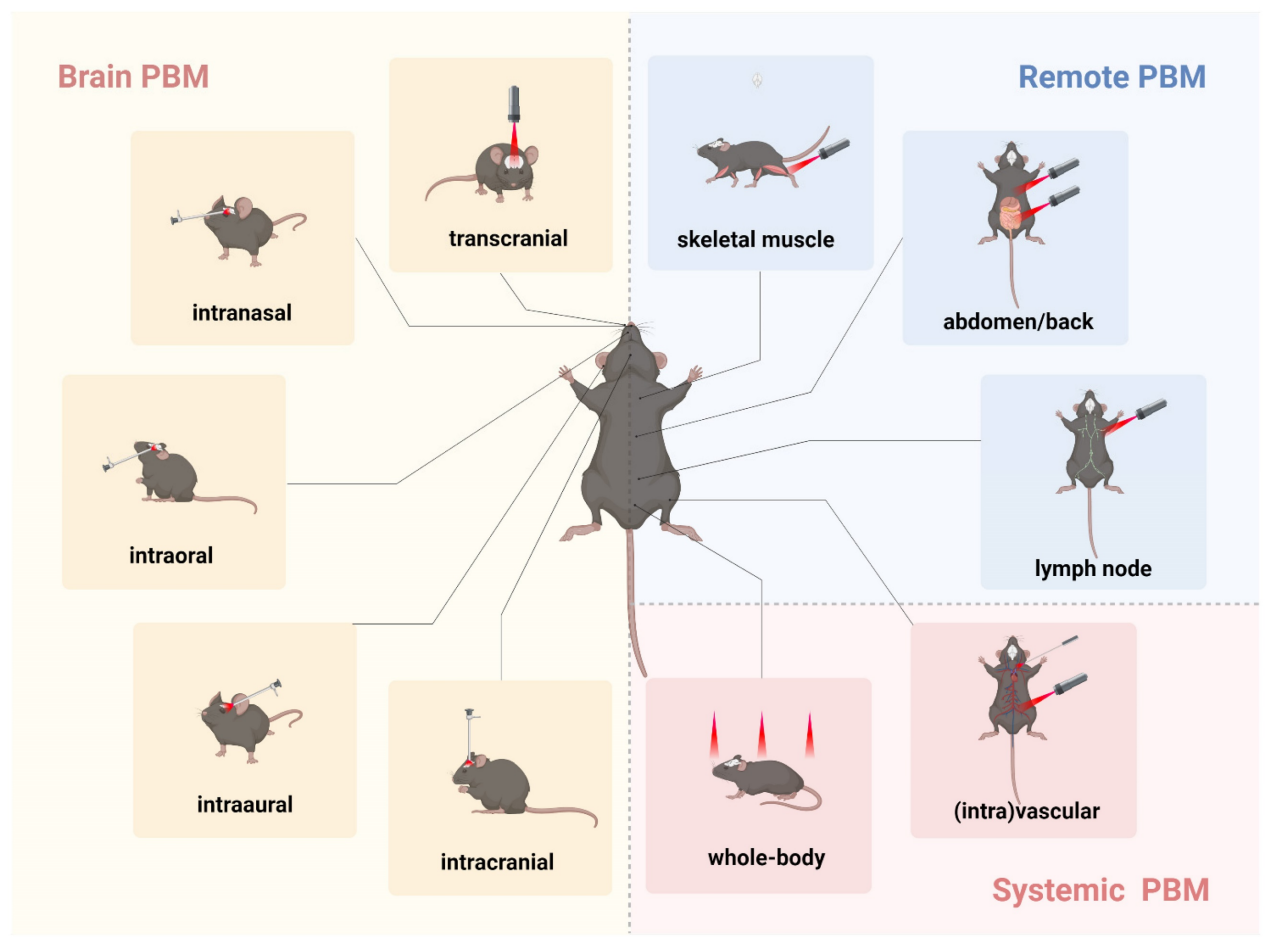
** Different transmissions of photobiomodulation (PBM) in animal models.** The various transmissions used in rodent models in PBM studies are as follows. ***Brain PBM (yellow)*:** transcranial, intranasal, intraoral, intraaural, and intracranial transmissions. ***Remote PBM (blue)*:** skeletal muscle, abdomen, back, and lymph node transmissions. ***Systemic PBM (red)*:** vascular, intravascular, and whole-body transmissions. Created using BioRender.com.

The potential of PBM as a treatment for depression lies in its capacity to regulate neural activity, reduce inflammation, and promote neuroplasticity, thereby alleviating depressive symptoms. However, the direct transmission of light through scalp tissue and cranial bones is likely not the sole contributor to PBM's beneficial effects on brain function. One hypothesis suggests that light delivery to multiple tissues may confer systemic benefits to the brain, a phenomenon known as indirect or abscopal effects 151. Consequently, research on PBM's impact on brain function can generally be categorized based on the site of light application within the body: brain PBM, remote PBM, and systemic PBM **(Figure 4)**.

### 6.1 Brain PBM

By directing light to the brain, tPBM directly influences brain function. Notably, transcranial approaches are currently the most extensively studied method in brain PBM research. However, delivering therapeutic or biostimulatory doses effectively to deeper brain structures, such as the limbic system and brainstem, presents a significant challenge 40. Due to the exponential attenuation of light intensity as it penetrates brain tissue, overexposure of superficial tissues is often necessary to deliver an adequate dose to deeper areas. Consequently, in addition to direct application to the skull, the literature has proposed several noninvasive routes for brain PBM, including intranasal 152, intraaural 153, and intraoral approaches 154, as well as invasive intracranial methods **(Figure 4)** 155.

To overcome certain limitations of tPBM, intranasal PBM has been suggested as an alternative for targeting prefrontal and select limbic regions. Intranasal PBM has traditionally been used to treat a range of brain conditions, including cerebral thrombosis, cerebrovascular diseases, mild cognitive impairment, AD, PD, post-stroke depression, and insomnia, with promising clinical outcomes 156. Recently, intranasal PBM devices designed for deep nasal cavity placement have been developed, aiming to deliver adequate light energy to deeper central nervous system structures and complement existing portable intranasal systems 157. Pitzschke *et al.* compared the penetration efficacy of 670 nm and 810 nm wavelengths into cerebral tissue using transcranial and transsphenoidal approaches, finding that transsphenoidal delivery of 810 nm light was more effective 154. As a potential treatment for depression, brain PBM enhances brain function, improves mood, and alleviates depressive symptoms by modulating neural activity and promoting neuroplasticity 49.

### 6.2 Remote PBM

Unlike brain-targeted PBM, remote PBM indirectly influences brain function by illuminating different body regions, such as skeletal muscles, abdomen, back, lymph nodes, and pain sites **(Figure 4)**. This approach induces whole-body physiological changes that ultimately impact brain function. Studies have shown that 810 nm PBM (1.2-36 J/cm^2^) applied to the head, neck, dorsal root ganglion, and ipsilateral right hind paw modulates neural pathways associated with pain perception and processing, offering potential as a non-invasive treatment for pain and other neurological conditions 158. Recent research has indicated that 635 nm PBM (10 J/cm^2^) applied to axillary lymph nodes improved cognitive function in AD mice by promoting CD4^+^ T cell infiltration into brain parenchyma, modulating immune responses, and enhancing neurogenesis 102. Additionally, PBM targeting the gut microbiota in amyloid-beta-induced AD mouse models, with 630 nm and 730 nm light (100 J/cm^2^) applied to the abdomen, altered microbiota diversity and richness, reduced amyloidosis and tau phosphorylation, and significantly improved cognitive function 159. Brain-gut PBM shows promise for translation from preclinical studies to clinical trials for AD. In a daily regimen, PBM applied to the head and abdomen in an AD mouse model demonstrated neuroprotective effects, with preliminary human trials suggesting that PBM therapy is safe and improves cognitive function in patients with mild-to-moderate AD 160.

Additional studies have demonstrated that applying 670 nm remote PBM (18 J/cm²) to the abdomen, back, or hindlimbs exerts neuroprotective effects by enhancing resistance to MPTP-induced neurotoxicity in a mouse model of PD 161. Remote PBM may stimulate circulating immune cells, stem cells, mitochondria, and the cardiovascular and lymphatic systems. In an experimental autoimmune encephalomyelitis model, 670 nm remote PBM (5 J/cm^2^) applied directly to the shaved dorsal surface of mice targeted the spinal cord, modulating immune responses and reducing central nervous system inflammation 162. Another potential mechanism underlying remote PBM's effects may involve its impact on blood platelets, potentially increasing systemic ATP availability 163. Reduced mitochondrial respiratory rates in blood platelets have been observed in patients with depression and are suggested to contribute to its etiology 164. Thus, remote PBM may enhance mitochondrial function and ATP availability throughout the body, producing antidepressant effects similar to those observed with tPBM.

### 6.3 Systemic PBM

In addition to remote application, systemic PBM therapy using laser or LED devices has demonstrated protective effects on the nervous system in multiple animal studies 99. This approach involves either direct whole-body exposure or achieving systemic effects through vascular or intravascular routes **(Figure 4)** 165. In one study investigating PBM's effectiveness in treating PD, researchers shielded the heads of mice with aluminum foil, thereby limiting light exposure to the body 166. While they observed significant neurocognitive improvements with head exposure, they also noted statistically significant, though less pronounced, benefits when the head was shielded. In a rat stroke model, direct exposure to 710 nm LED light positioned above the animal cages substantially enhanced immune cell function, reduced microglial activation, decreased brain infarct size, and improved neurological outcomes 167. Whole-body LED PBM at 1072 nm further demonstrated cognitive enhancement and reduced Aβ plaque accumulation in transgenic mice 168.

Recently, Thor Photomedicine (Chesham, Bucks, UK) introduced the NovoThor LED whole-body light pod, which utilizes 660 and 850 nm wavelengths for whole-body treatment in humans 169. This non-invasive approach could potentially be beneficial for both preconditioning and post-conditioning in various brain disorders. Collectively, systemic PBM has shown neuroprotective effects by enhancing systemic circulation, reducing inflammation, and modulating physiological responses, making it a promising candidate for alleviating depression.

## 7. Safety and tolerability

PBM has been determined to be generally safe and easily tolerated. Not all studies reported adverse events, and among those that did, the incidence was minimal, with most effects being mild and transient 170. The most commonly reported adverse effects included insomnia, irritability, sensory illusions, and abdominal bloating 171. The primary complaint was fatigue, accompanied by a headache and a dry mouth. These adverse effects were mild and transient, typically resolving within a day following treatment 172. The risk of thermal damage from PBM is considered minimal and mainly limited to the skin, which has the highest light absorption capacity. A recommended safety measure is to use pulsed wave during intense light exposure to prevent neurological deficits and histopathological brain damage 45. However, it is essential to acknowledge that the use of laser devices carries an inherent risk of retinopathy if improperly operated, particularly if the light beam is directed onto the macula.

## 8. Therapeutic applications of PBM in depression

### 8.1 Animal models

**Figure 5:**
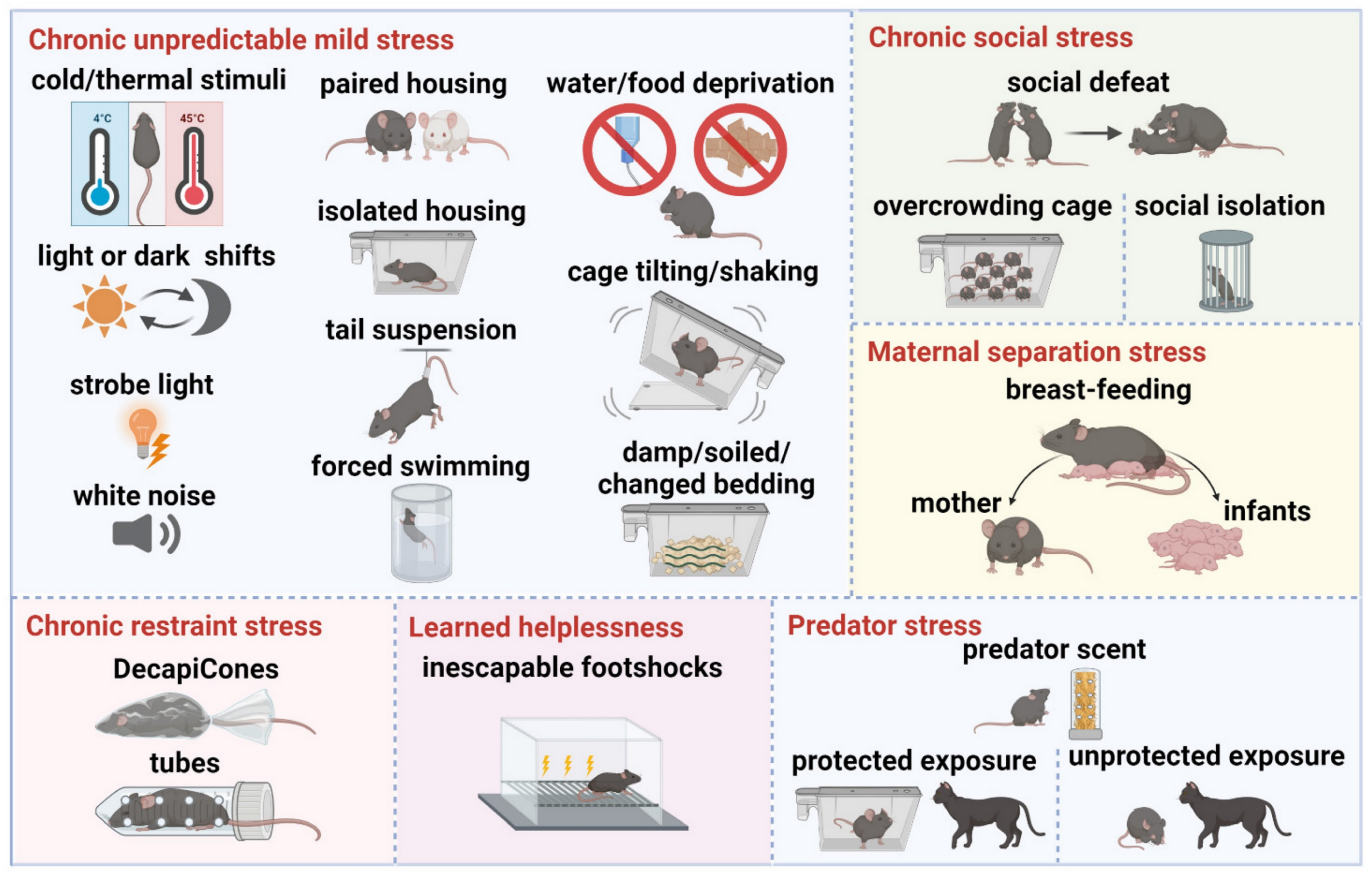
** Depression animal models of stress paradigms. *Chronic unpredictable mild stress*:** cold/thermal stimuli, light or dark shifts, strobe light, white noise, paired housing, isolated housing, tail suspension, forced swimming, water/food deprivation, cage tilting/shaking, damp/soiled/changed bedding. ***Chronic restraint stress*:** DecapiCones, tubes. ***Chronic social stress*:** social defeat, overcrowding cage, social isolation. ***Maternal separation stress*:** early weaning, separation from mother and infants. ***Learned helplessness*:** inescapable footshocks. ***Predator stress*:** predator scent, protected exposure, unprotected exposure. Created using BioRender.com.

The various stress paradigms employed in current animal models of depression simulate different symptoms of the disorder, facilitating researchers' understanding of its underlying mechanisms and supporting the exploration of potential therapeutic interventions **(Table 1)**. These paradigms involve stressors designed to induce depressive-like behaviors in rodents. The main stress paradigms include chronic unpredictable mild stress, chronic restraint stress, chronic social stress, maternal separation stress, learned helplessness, and predator stress **(Figure 5)**.

Recent studies on PBM therapy have shown promising results in treating depression in various animal models, suggesting its potential as a therapeutic intervention for these conditions. For instance, one study proved that tPBM using an 810 nm laser at a 10 Hz pulse frequency and a fluence of 36 J/cm² significantly improved neurobehavioral recovery and exhibited antidepressant effects in mice with TBI, compared to continuous wave and 100 Hz pulse frequency treatments, underscoring the importance of optimizing pulse frequency in PBM protocols 65. Another study further demonstrated that tPBM at 810 nm, using the same 10 Hz frequency but a lower fluence of 8 J/cm², effectively reduced anxiety and depression in a mouse model by modulating serotonin and NO levels in the cerebral tissue 54. These findings highlight the potential of pulse frequency adjustments in enhancing PBM's antidepressant effects. Similarly, tPBM with an 830 nm laser at a fluence of 15.28 J/cm² exhibited significant anti-depressive and antioxidant benefits in a rat model of depression, suggesting the therapeutic advantages related to specific wavelengths and fluence levels 173.

Supporting this evidence, a study revealed that tPBM at a 10 Hz frequency with an 810 nm laser at a fluence of 14.4 J/cm² improved depressive-like behaviors in rats subjected to chronic mild stress, with efficacy comparable to that of the antidepressant Citalopram and superior to red light PBM treatments 145. Such findings emphasize tPBM's potential as an alternative or adjunct therapy to pharmacological approaches. In another application, tPBM with an 804 nm laser at 80 mW effectively alleviated reserpine-induced depression in rats by enhancing activity levels and normalizing electrophysiological patterns, offering potential benefits for treatment-resistant cases 174. In addition, studies employing 808 nm tPBM at a fluence of 41.4 J/cm² significantly reduced depression-like behaviors in mice by boosting ATP production and CCO activity in the prefrontal cortex, emphasizing the therapeutic value of targeting specific brain regions 47.

In models of post-traumatic stress disorder, tPBM with an 808 nm laser at a fluence of 3 J/cm² effectively managed symptoms in rats exposed to underwater trauma by modulating neuronal activity in the hippocampus and amygdala, enhancing ATP production and early gene expression 175. This protocol also shows potential for preventing comorbid conditions, such as depression, through early intervention. Early tPBM treatment with an 808 nm laser at 3 J/cm² was shown to prevent oligodendrocyte dysfunction and depression-like behaviors in rats exposed to early life adversity 176. Complementary studies using a 635 nm tPBM at a fluence of 2 J/cm² in a chronic mild stress model upregulated glutamate transporter-1 expression, thereby enhancing glutamate clearance—a mechanism potentially crucial for resilience against stress-induced depression 177. Extending these insights, tPBM at an 808 nm wavelength with a fluence of 3 J/cm² effectively improved anxiety and depressive symptoms in TgF344 rats by attenuating neuronal damage, reducing neuroinflammation, and improving mitochondrial function, suggesting potential pathways for mitigating depressive symptoms at the cellular level 178.

Additionally, in noise-induced stress models, tPBM with 810 nm (8 J/cm², 10 Hz) led to significant reductions in anxiety and depressive behaviors, likely mediated by increases in BDNF levels and reductions in oxidative stress and inflammation 179. Combining 810 nm tPBM at a fluence of 33.3 J/cm² alongside Coenzyme Q10 supplementation produced significant neuroprotective effects, reducing neuroinflammation, oxidative stress, and apoptosis in depression models, indicating potential for integrated treatment approaches 92. Innovative applications of brain-gut PBM therapy using dual wavelengths (660 nm LED at 2.38 J/cm²; 850 nm LED/ laser at fluences of 3.33 J/cm² and 1.59 J/cm²) have shown promise in restoring cognitive function in chronically stressed mice by modulating Sirt1 levels and reducing neuroinflammation 180.

These findings illustrate PBM's therapeutic effects extend beyond depressive symptom reduction to encompass neuroprotection and cognitive resilience. Key factors for clinical optimization include selecting appropriate wavelengths, fluence levels, and pulse frequencies that maximize therapeutic effects. Current studies suggest that 808-830 nm wavelengths, particularly with 10 Hz pulsed settings and fluences between 8-36 J/cm², are effective in various animal models. However, further research is essential to refine these parameters, clarify mechanisms of action, and evaluate potential benefits of combination therapies, such as adjunct antioxidant treatments. These steps will be critical to firmly establish PBM as a reliable, non-invasive treatment option in clinical settings for managing mood disorders.

### 8.2 Human subjects

Recent studies underscore the effectiveness of PBM therapy in alleviating depression symptoms in humans, highlighting its potential as a promising treatment option with minimal side effects. Multiple studies support PBM's ability to significantly reduce depressive symptoms. For instance, one study found that tPBM at 810 nm and a fluence of 60 J/cm^2^ notably decreased depression and anxiety symptoms in individuals with MMD, with sustained improvements observed at both 2 and 4 weeks post-treatment 43. Another study reported that tPBM at 808 nm with an 84 J/cm² fluence led to a marked reduction in depression scores, reinforcing the efficacy and safety of PBM in treating mood disorders 135. In a related study, a multi-Watt tPBM approach with dual wavelengths (810/980 nm) and a fluence of 55 to 81 J/cm^2^ demonstrated substantial reductions in depressive symptoms in patients with comorbid depression, with 92% showing a positive response and 82% achieving remission 181.

Further reinforcing its clinical utility, PBM's sustained benefits are exemplified in a long-term case study. This study employed tPBM at 830 nm with fluences of 49.8 J/cm^2^ and 59.8 J/cm^2^, significantly alleviated depression and anxiety-related symptoms in a patient with both anxious depression as well as Takotsubo cardiomyopathy over a 31-month period 171. Moreover, PBM's applications extend beyond mood disorders, as unilateral tPBM at 810 nm and 60 J/cm² showed a significant reduction in opioid cravings, depression, and anxiety, with a notable effect size of 0.73 over sham treatments 182. Targeted PBM therapy, especially when applied to the prefrontal cortex via transcranial and intranasal methods, has proven effective in emotional regulation for MDD, thereby presenting a targeted approach for clinical settings 157.

However, recent studies have raised considerations for personalized PBM applications. For instance, a study found that older patients received less energy in targeted brain regions due to thicker extracerebral tissue, suggesting that age-related changes impact treatment efficacy. For these individuals, an 810 nm wavelength with a 3 J/cm² fluence produced the most effective results, underscoring the need for age-specific dose adjustments 183. Similarly, a study using a 945 nm wavelength and 9.35 J/cm² fluence significantly enhanced brain activity and lowered depression and anxiety in university students, indicating tPBM's adaptability to different demographic needs 184. Another study observed that tPBM at 810 nm with 21 J/cm² also improved cognitive function in the frontal lobe while reducing depressive symptoms in elderly individuals with non-amnestic mild cognitive impairment, highlighting its potential for managing mood and cognitive symptoms in aging populations 185.

While PBM's broad effectiveness is well-supported, some limitations indicate the need for precise dosing. For example, studies have shown that very low levels of irradiance and energy were ineffective in alleviate depression symptoms in MDD patients, indicating that a minimum effective dose is essential for achieving clinical benefits 186. Additionally, in cases of chronic schizophrenia, tPBM at 630 nm and 810 nm with a fluence of 144 J/cm² did not improve cognitive or psychotic symptoms but did show a temporary effect in alleviating anxiety and depression symptoms. This suggests that PBM may serve as a complementary therapy in complex psychiatric conditions, with potential benefits for relieving mood-related symptoms 187.

Extending beyond primary mood disorders, PBM therapy has also demonstrated benefits in managing depression-related symptoms associated with other conditions. For example, PBM applied to the abdomen and thighs (191.4 J/cm² and 86.4 J/cm², respectively) at 904 nm helped reduce fatigue, depression, and pain in young adults with inflammatory bowel disease, offering initial support for its feasibility in chronic conditions with associated mood disturbances 188. Similarly, PBM at 660 nm and 850 nm with a fluence of 3 J/cm² targeted to the back and thighs alleviated depressive symptoms in patients with lower back pain, while PBM at 632.8 nm with 37.5 J/cm² applied to neck vessels and auricular points effectively reduced depression symptoms among individuals with alcohol addicts 189, 190. Additionally, 808 nm laser-based acupuncture has proven beneficial for mild to moderate depression, providing an alternative treatment option with significant therapeutic potential 172, 191.

In summary, PBM therapy demonstrates a significant capacity for managing depression symptoms across various conditions and demographics. Optimal parameters, as identified in current studies, suggest wavelengths in the range of 808-830 nm, fluences of 60-84 J/cm² for general use, and lower fluences (around 3 J/cm²) for specific demographic adjustments. Age-related adjustments and targeted applications to the prefrontal cortex or other mood-regulating regions may further improve treatment efficacy. However, establishing a minimum effective dose and personalizing treatment protocols are essential for maximizing PBM's clinical benefits. Future research is recommended to refine these parameters and explore PBM as a viable option for treating mood disorders in diverse populations.

Based on recent advances in both animal and human studies, PBM therapy demonstrates significant promise in alleviating depression and anxiety symptoms across diverse models and patient populations. Animal models reveal that PBM, particularly within specific wavelength and fluence ranges, effectively reduces depressive-like behaviors and enhances mitochondrial and neurochemical function. These preclinical results provide a foundation for clinical research, which has shown promising outcomes in human studies, especially in alleviating depression and anxiety symptoms with tailored parameters. Moreover, the consistent efficacy observed in both animal and human studies emphasizes the importance of targeting treatment to specific brain regions, such as the prefrontal cortex, as the primary target area for tPBM treatment of depression and anxiety symptoms. Focusing on this region has proven effective in enhancing ATP production, CCO activity, and emotional regulation. For broader applications, such as managing mood symptoms associated with chronic conditions, PBM has also shown benefits when applied to non-cranial regions like the abdomen, thighs, and neck vessels. These sites offer therapeutic advantages for depressive symptoms in conditions such as inflammatory bowel disease, chronic pain, and addiction, extending the utility of PBM beyond primary mood disorders. Moving forward, the integration of personalized dosing and targeted applications holds significant potential to establish PBM as a viable and non-invasive therapeutic option for treating mental health conditions more broadly.

## 9. Limitations and future directions

Despite advancements in laboratory and clinical research, achieving effective transmission of light energy to reach brain tissue through the scalp and skull remains a considerable challenge in PBM application. To maximize light penetration in targeted areas, hair should be either shaved or parted, and light should be applied directly onto the skin. Moreover, research on neuroprotection using remote PBM remains in its early phase, requiring further comprehensive studies. We suggest using PBM aimed at the abdomen to reduce the local inflammation, potentially by activating the microbiome-gut-brain axis, the HPA axis, or directly affecting mucosal neurons. Additionally, targeting the large muscles of the legs could reduce the inflammatory response and alleviate peripheral fatigue in inflammatory bowel disease, ultimately easing central symptoms such as depressive disorders and central fatigue. This effect could be achieved either through its direct local impact or by modulating the neuroimmune system.

This review acknowledges certain limitations regarding the characteristics of the studies analyzed, which encompass a broad range of methodologies that often lack specificity and sometimes fail to provide detailed parameters. Additionally, the criteria for inclusion of participants varied widely among the research. Some studies on tPBM exclusively included individuals with primary MDD, while others assessed depression levels among patients with broader neurological conditions. Consequently, additional *in vivo* and *in vitro* studies with expanded sample sizes are required to support consistency in treatment protocols. Optimal PBM parameters—including stimulation sites, frequency and duration of treatments, device type, wavelength, irradiance, and fluence—can improve the therapy's safety and effectiveness, as well as standardize its application to minimize adverse effects. Once optimal dosages are identified, researchers can design extensive randomized controlled trials to evaluate the therapeutic impact.

Recent clinical studies registered on ClinicalTrials.gov suggest that PBM holds considerable promise for treating various forms of depression, including childhood, adolescent, geriatric, perinatal, and opioid-induced depression, as well as MDD, bipolar disorder, and attention-deficit/hyperactivity disorder. Focus areas include enhancing cerebral blood flow, improving emotional state, reducing fatigue, and addressing symptoms like anxiety, stress, and sleep disturbances. In recent years, there has been a growing emphasis on personalizing phototherapy regimens to suit individual needs, considering variables such as genotype, sleep patterns, and light sensitivity. By customizing the combination of light dose, exposure time, and spectrum, therapeutic efficacy can be enhanced while minimizing side effects, thereby ensuring the long-term safety and effectiveness of these therapies in clinical settings. Additionally, a growing number of studies have explored the potential benefits of combining phototherapy with other treatment modalities, such as behavioral therapy and pharmacotherapy. The combination of phototherapy and antidepressants may prove to be more effective than either treatment alone. Simultaneously, efforts are underway to develop more portable and user-friendly phototherapy devices, including wearable LED systems suitable for use at home or in workplace settings, thus increasing accessibility to continuous treatment.

## 10. Conclusions

PBM technology offers a promising approach for counteracting the cellular and molecular dysfunctions associated with energy depletion in depressive disorders. Devices equipped with optimized settings can enhance PBM's efficacy and reliability while minimizing potential side effects. Combining PBM with conventional therapies may provide substantial symptom relief, representing a promising treatment option for patients with MDD. However, current research on PBM's impact on patients with treatment-resistant depression faces substantial challenges, including the need for precise and standardized diagnostic criteria for depression and consistency in PBM treatment parameters. Looking forward, PBM has the potential for wider societal use, whether in clinical settings or home applications through laser and LED devices, and may become a powerful tool for alleviating depression and related neurological symptoms. In conclusion, light-based therapies are likely to become increasingly relevant in cognitive neuroscience and psychiatry, with PBM serving as an essential tool for enhancing public health and managing psychiatric disorders.

## Figures and Tables

**Table 1:** Comparison of stress-induced depression animal models.

Stressor type	Brief description	Human Equivalent	Ref.
Chronic unpredictable mild stress	Repeated cold stress, thermal stimuli, intermittent air puff, strobe light, intermittent illumination, light or dark cycle shifts, white noise, cage tilting/shaking, no/damp/soiled/changed bedding, water/food deprivation, paired housing, isolated housing, tail suspension, forced swimming.	Chronic stress/anhedonia/anxiety/despair; Reduced interest/movement; Heightened reactivity to sudden unexpected stimuli; Highly sensitive to environmental conditions; Various prolonged, unpredictable disturbances in daily life.	192
Chronic restraint stress	Immobilization/restraint stress; Placed in a plastic cylinder/tube/small container.	Physical restraint/confinement; Behavioral despair/anhedonia/anxiety; Restraining or handling uncomfortable stimuli; Exposure to inescapable situations/place; Daily unpleasant situations at work or abusive jobs.	193
Chronic social stress	Social defeat/conflict; Resident-intruder paradigm; Visual stress/olfactory stress/physical contract; Social isolation; Isolation stress/reared in small individual cages; Uncertain social environment; Overcrowding/frequent changes in cage mates or locations.	Social stress/disruption/deficits/avoidance/deprivation/isolation/withdrawal; Anhedonia/despair/anxiety/loneliness; Agonistic/stereotyped/submissive behaviors; Dysfunctional social behavior/bullying and psychological abuse; No peer interaction/less contact/reduction of enthusiasm/reduced shoaling; Social instability stress/unstable social hierarchy; Passive coping with inescapable stress; Exposure to open unprotected space/inescapable place.	194-196
Maternal separation stress	Maternal separation; Early weaning/social isolation environmental stress.	Social avoidance/despair/anxiety; Parental negligence/childhood trauma/growing up in poverty/early life stress; Predictable or unpredictable social stressors in juvenile period; Postpartum depression/changes in maternal behavior.	197, 198
Learned helplessness	Inescapable electric foot-shock; Continuous involuntary movement; Predictable physical stressor.	Hopelessness/anhedonia; Social avoidance/withdrawal/reduced escape behavior; Symptoms of traumatic stress; Unresolvable situation or problem.	199
Predator stress	Predator scent; Protected exposure; Unprotected exposure.	Anxiety/psychosocial stress; Symptoms of post-traumatic stress; Fear conditioning.	200

## Abbreviations

AD: Alzheimer's disease
ATP: adenosine triphosphate
BDNF: brain-derived neurotrophic factor
Ca²⁺: calcium ion
CAMP: cyclic adenosine monophosphate
CBF: cerebral blood flow
CCO: cytochrome c oxidase
eNOS: endothelial nitric oxide synthase
IL: interleukins
LED: light-emitting diode
MMP: membrane potential
NF-κB: nuclear factor-κB
NIR: near-infrared
NO: nitric oxide
PBM: photobiomodulation
PD: Parkinson's disease
ROS: reactive oxygen species
TBI: traumatic brain injury
tPBM: transcranial photobiomodulation
VEGF: vascular endothelial growth factor
